# Functional Outcomes of Children Identified Early in the Developmental Period as at Risk for ASD Utilizing the The Norwegian Mother, Father and Child Cohort Study (MoBa)

**DOI:** 10.1007/s10803-020-04539-8

**Published:** 2020-05-18

**Authors:** Nina Stenberg, Synnve Schjølberg, Frederick Shic, Fred Volkmar, Anne-Siri Øyen, Michaeline Bresnahan, Britt Kveim Svendsen, Stephen von Tetzchner, Nina Torheim Thronæs, Suzanne Macari, Domenic V. Cicchetti, Katarzyna Chawarska, Pål Suren, Roald A. Øien

**Affiliations:** 1 Oslo University Hospital, Oslo, Norway; 2 Norwegian Institute of Public Health, Oslo, Norway; 3 Department of Education, UiT – The Arctic University of Norway, PB 6050, 9037 Tromsø, Norway; 4 School of Medicine, Child Study Center, Yale University, New Haven, USA; 5 Center for Child Health, Behavior and Development, Seattle Children’s Research Institute, Seattle, WA USA; 6 Department of Pediatrics, University of Washington, Seattle, WA USA; 7 Mailman School of Public Health, Columbia University, New York, USA; 8 Nic Waals Institute, Lovisenberg Hospital, Oslo, Norway; 9 Department of Psychology, UiO, Oslo, Norway

**Keywords:** MoBa, Norwegian mother, Father and child cohort study, Cognition, Language, Autism spectrum disorders, Vineland, ADOS, Screening, Early identification

## Abstract

Early identification of autism spectrum disorder (ASD) is regarded as crucial for swift access to early intervention and, subsequently, better outcomes later in life. However, current instruments miss large proportions of children who later go on to be diagnosed with ASD, raising a question of what these instruments measure. The present study utilized data from the Norwegian Mother, Father, and Child Cohort Study and the Autism Birth Cohort study to explore the subsequent developmental and diagnostic characteristics of children raising developmental concern on the six-critical discriminative item criterion of the M-CHAT (DFA6) at 18 months of age (N = 834). The DFA6 identified 28.8% of children diagnosed with ASD (N = 163), but 4.4% with language disorder (N = 188) and 81.3% with intellectual disability (N = 32) without ASD. Scoring in the «at-risk» range was associated with lower IQ, impaired functional language, and greater severity of autism symptoms whether children had ASD or not.

## Introduction

Early identification of autism spectrum disorder (ASD) is regarded as crucial for prompt access to early intervention, and subsequently, better outcomes later in life (Fernell et al. 2013; Mandell et al. 2005; Zwaigenbaum et al. 2015). Recent studies have revealed that only a small proportion of young children, between 18 and 36 months of age, eventually diagnosed with an autism spectrum disorder (ASD) are captured by screening instruments (Guthrie et al. 2019; Øien et al. 2018b; Stenberg et al. 2014; Surén et al. 2019). Thus, it is essential to determine if children who are diagnosed early show different behavioral characteristics compared to children who are missed. A better understanding of these characteristics may allow providers to identify early interventions that are best suited to a children’s specific needs (Fernell et al. 2013).

Currently, a range of autism-specific screening measures exist that probe for early developmental and behavioral delays or deviancies. The Modified Checklist for Autism in Toddlers (M-CHAT) (Kleinman et al. 2008; Robins et al. 2001) and subsequent M-CHAT Revised with Follow-Up (M-CHAT-R/F) (Robins et al. 2014) are currently the most studied screening instruments for children younger than 36 months. However, studies reveal that the M-CHAT fails to identify the majority of 18-month-olds who are later diagnosed with ASD (Guthrie et al. 2019; Øien et al. 2018a; Stenberg et al. 2014; Sturner et al. 2017). One possible cause of the discrepancy in findings may be that measures implemented in the M-CHAT-R/F aimed to reduce false positives, not to increase the identification of false negatives (Øien et al. 2018a; Stenberg et al. 2014). Additionally, many studies using the M-CHAT and M-CHAT-R/F in large samples only validate the screening results with a full assessment of the screen positives for ASD (Chlebowski et al. 2013; Kleinman et al. 2008; Pandey et al. 2008; Robins et al. 2001, 2014). As a result, these studies are unable to capture false negatives because few screen negatives are reassessed between 18 and 24 months of age, and even fewer are followed later to examine diagnostic status and developmental trajectories.

While the primary rationale for screening is to improve early identification and facilitate swift access to intervention, most screening-related efforts have had a limited effect on decreasing the age of diagnosis. Despite many parents expressing concerns when children are 15 months of age or younger (Chawarska et al. 2006), recent epidemiological studies report that the average age of diagnosis remains between 3 and 5 years (Baio 2012; Baio et al. 2018; Fountain et al. 2011; Mandell et al. 2010; Surén et al. 2019). Further research is needed to delineate the differences in early behavioral characteristics, developmental trajectories, and age of parental concern in early- versus late-diagnosed children with ASD in the general population.

It is essential to address the sparsity of knowledge regarding the variability in developmental and behavioral characteristics of 18-month-old children who are later diagnosed with ASD. A minority of these children meet the threshold to be considered as "at-risk” at 18 months based on screening instruments (Stenberg et al. 2014). It is particularly unclear how the phenotypic presentations of these “at-risk” children later diagnosed with ASD differ from those of autistic children who were not identified by gold-standard screening measures and who would hence have been considered as “low-risk” at 18 months (Beacham et al. 2018; Øien et al. 2018a). Increased knowledge of phenotypic differences associated with screening performance and outcomes is critical for addressing the challenges of identifying a more substantial proportion of children at 18 months that later go on to develop ASD. This is especially true for those children who are not identified by the currently used core symptom patterns expected at 18 months as concurrent or emerging early markers for ASD.

The current knowledge gap is related to the understanding of the association between broader behavioral and developmental characteristics of children at the time of screening, and later developmental outcomes in those identified prospectively with ASD. Because most screening studies are conducted in high-risk samples, less is known about the expression of children missed by screening instruments (i.e., children regarded as "low-risk" after initial screening but receiving a diagnosis later). Understanding which early behavioral and developmental symptoms characterize children who are missed in early screening provides a basis for improving ASD screening instruments and can substantially increase their efficiency in identifying children as early as 18 months of age who are on a path of being diagnosed with ASD. For example, some studies, such as studies of infant siblings of children with ASD, have found that early developmental characteristics (fine motor skills at six months) predicted the severity of ASD symptoms both at 18 and 36 months of age (Iverson et al. 2019). Other studies have found that combining a screening instrument with a general developmental checklist improved the prediction of a later ASD diagnosis (Beacham et al. 2018).

Finally, it is crucial to understand whether screening instrumssents can identify children who are later diagnosed with neurodevelopmental disorders other than ASD (i.e., false positives). Children with more advanced language skills and IQ within the average range might make some toddlers appear more typical. These toddlers often do not meet the threshold for parental or professional concern and, because little is known about the heterogeneity of predictive symptoms of ASD, they are more challenging to identify as at-risk for ASD (Øien et al. 2018a; Salomone et al. 2015; Surén et al. 2019). However, there may be other symptom patterns associated with the development of ASD that have yet to be explored in screener development (Chawarska et al. 2014). It is crucial to understand more about the developmental and behavioral characteristics that predict ASD diagnosis and ASD subgroup identification so that we may tailor screening instruments and increase the performance of early identification. This could enable researchers to identify different subgroups of children later diagnosed with ASD, but who do not early show the types of symptoms enlisted in the current screening instruments.

The aims of the current study are to:

1. compare child characteristics at the time of clinical diagnostic assessment and relate those to the presence (at-risk) or absence (low-risk) of core ASD symptoms at 18 months, i.e., including both children with early symptoms who were later diagnosed with ASD (true positives), and children with no symptoms, but who were still diagnosed with ASD later (false negatives).
2. compare developmental characteristics at the clinical assessment of children *with* and *without ASD* depending on whether they rated *above* or *below* the threshold for concern on core ASD symptoms at 18 months. The four groups (true positives, true negatives, false positives, and false negatives) are compared on measures of (a) cognition (IQ), (b) functional language (Vineland Adaptive Behavior Scales; Communication Domain), and c) ASD symptom severity (ADOS CSS) at the time of clinical assessment.
3. examine whether the core symptoms (as measured by the screening instrument) for ASD are specific to ASD, or whether measures screening for these symptoms also characterize children with other neurodevelopment diagnoses, i.e., detailing the diagnostic outcome of false positives and false negatives.

## Methods

### Participants

The present study utilizes data collected in the Autism Birth Cohort study (ABC study) (Stoltenberg et al. 2010). The ABC Study is a sub-study of the Norwegian Mother, Father, and Child Cohort Study (MoBa) (Magnus et al. 2016) aiming to identify all ASD cases within MoBa (Surén et al. 2019). MoBa is a national prospective general population pregnancy cohort conducted by the Norwegian Institute of Public Health. Participants were recruited from all over Norway between 1999 and 2008. The women consented to participation in 41% of the pregnancies and included 114, 552 children, 95,000 mothers, and 75,200 fathers (Magnus et al. 2016). Parents who agreed to participate in MoBa and the ABC Study signed an informed consent form, and the study was approved by the Regional Committee for Medical and Health Research Ethics South East. MoBa data version 9 was used.

The mothers completed questionnaires during pregnancy and at regular intervals after birth, at 6, 18, and 36 months as well as at 5, 7, 8, and 14 years (still ongoing). The MoBa questionnaires include items on child development and behavior as well as health, diet, socioeconomic factors, and other factors relevant to child and parental health. Parents completed the questionnaires independently, without assistance from a health care professional. The clinical assessments in the ABC study started in 2005 and ended in 2012, finalizing the assessment of 1,033 children. Since the end of the clinical assessments, the ABC sample is followed through yearly linkage to the Norwegian Patient Registry (NPR), a national registry containing information about all discharge diagnoses on children who were referred to specialized health services. The study has information about discharge diagnosis from 2008 through 2015.

### Recruitment of the Sample

Multiple methods identified potential cases of ASD seen in the ABC study clinic, including MoBa questionnaire information (at child age 3, 5, and 7 years), referrals (parental or professional), and annual linkages to the Norwegian Patient Register (NPR). The clinical study focused primarily on assessing 3-year-olds and utilized six questionnaire-based selection criteria from the 3-year-old questionnaire in MoBa (Q3yr). The selection criteria cast the screening net wide to ensure that all children on the path to a diagnosis of ASD would be captured.

Families endorsing any of the following six specific criteria were invited for a clinical assessment: (Criterion 1) Families of children scoring at or above a cutoff of 12 on the Social Communication Questionnare (SCQ) embedded in Q3yr (see details on the study-specific screening procedures in Stoltenberg et al. 2010, pp.677–678). (Criterion 2) Families of children endorsed on all nine repetitive behavior items on the SCQ. (Criterion 3) Families who reported language delay under the “health problem” section in Q3yr and referred to a language specialist. (Criterion 4) Families who reported "yes" on autism or autistic traits under the "health problem” section in Q3yr, and/or “yes” on autism/Asperger syndrome in Q5yr or Q7yr “health problem” section. (Criterion 5) Families who reported concerns about the child’s lack of interest in playing with other children under the “concern about the child” section. (Criterion 6) Families who reported that others (e.g. family, day-care staff, and well-baby nurses) had expressed concern about the child's development. In addition to the specific criteria based on the 3-year-old questionnaire, both professionals and parents could refer a child with suspected ASD to the study if the family/child were participating in MoBa and the child was born in the same time interval as the questionnaire-recruited children in the study.

Among the 1033 children clinically assessed in the ABC study, 665 were children who met one or more of the high-risk criteria listed above. The remaining 368 comprised the random control group and were drawn from the same cohort in a nested case–control design. For details on the ABC study criteria and results, see (Stoltenberg et al. 2010) and (Surén et al. 2019). After the research clinic ended in 2012, case ascertainment has continued through annual linkage to the NPR. At the last linkage in 2015, only 56 ASD cases were detected through the screening effort and clinical assessments. The vast majority of children with ASD were detected through the registry linkage after the clinical study had ended (Surén et al. 2019).

### Study Sample

This study takes advantage of the data collected in the ABC study and focuses primarily on the early developmental history of children who were diagnosed with ASD either in the ABC clinic or later through NPR linkage (the last linkage was done in 2015). The 18-month questionnaire in MoBa contained the complete M-CHAT checklist and was completed long before the ABC study started, and no criteria from this questionnaire were used in the ABC study. Children included in the final study sample had parents who (a) participated in the ABC study and returned the 18-month questionnaire, and (b) completed all of the six most discriminative items from the M-CHAT (Robins et al. 2001). The six discriminative items have better psychometric properties compared to the full M-CHAT in terms of sensitivity and specificity in identifying children with ASD based on their early symptom patterns (Kleinman et al. 2008; Øien et al. 2018a; Pandey et al. 2008; Stenberg et al. 2014). Totaling 834 children, the sample consisted of 539 with potential ASD (81.1% of the potential ASD assessed in the clinic) and 295 random controls (80.2% of controls assessed in the clinic). 547 children were males (65.6%), and 163 were diagnosed with ASD through ABC's clinical assessment or linkage to NPR.

### Clinical Diagnosis

ASD diagnoses: The children were assessed in the ABC study clinic by a research-trained team of experienced clinicians (specialists in clinical psychology or child psychiatry). Children were rendered a best-estimate diagnosis according to the Diagnostic and Statistical Manual of Mental Disorders, Fourth Edition, Text Revision (DSM-IV-TR) utilizing all available information from the clinical assessment. ASD symptoms were evaluated using the Autism Diagnostic Interview-Revised (ADI-R) and the Autism Diagnostic Observation Schedule-Generic (ADOS-G) (Lord et al. 2000). No information from MoBa questionnaires or NPR was available for the research team concerning individual children, i.e., they were blinded to health differences, developmental questions, parental concern, and selection criteria. A diagnostic summary meeting among the research staff after the data collection integrated all available clinical information, and the diagnosis was rendered, along with the certainty of the conclusion.

### Measures

#### Modified Checklist for Autism in Toddlers

The Modified Checklist for Autism in Toddlers (M-CHAT) (Robins et al. 2001) is the most widely used instrument for detecting early symptoms of ASD in young children (Ibaez et al. 2014), designed to be completed in the waiting room by a primary care provider (Robins et al. 2001). It has been recommended for use in toddlers at 18 months of age with a follow-up at 24 months (Bright Futures Steering Committee 2006). This instrument is the best-validated measure of early ASD signs and symptoms. Scores depend on either a six-critical discriminative item criterion (DFA6) or a total-23 item criterion (Total23). The six-critical item criterion is met when failing two or more of the six critical items, whereas the Total23 criterion demands a failure of three or more of any of the 23 items of the M-CHAT. It is recommended to do a follow-up phone interview of screen positives to reduce the number of false positives. Since the introduction of the M-CHAT in 2001, a revision of the checklist was released in 2014 (Robins et al. 2014), still used with the recommended follow-up interview to further reduce the number of false positives. The ABC study did not use any follow-up phone interviews in their design, as it is an epidemiological study, while the current study focuses on the relationship between early symptom patterns and later functional outcome both in children with and without ASD. Failing two or more of the six critical items at 18 months is treated as consistent reporting of core ASD symptoms (i.e., being at-risk). It is not an indication that such a child would be diagnosed with ASD this early, even if an experienced autism specialist evaluated the child at 18 months.

#### Cognitive Skills

Children's cognitive level was assessed with an appropriate developmental test, depending on age and cognitive level. As a general rule, the Stanford-Binet Intelligence Scales: Fifth Edition (SB-5) (Roid and Pomplun 2012) was the first choice for all children younger than 6 years of age. The SB-5 Brief IQ score (based on two routing-tasks from verbal and non-verbal domains) was calculated if the child did not complete all the subtests. If the child was not able to complete the routing tasks on the SB-5, a switch was made to the Mullen Scales of Early Learning (MSEL) (Mullen 1995). For children who were 6 years or older at clinical assessment, the Wechsler Abbreviated Scale of Intelligence (WASI) (Psychological Corporation 1999) was used, and the SB-5 was administered if the child was not able to complete WASI.

#### Communication Skills

The Vineland Adaptive Behavior Scales (VABS, 2nd edition) (Sparrow et al. 2005) is a parent interview assessing adaptive skills in four domains, communication, daily living, socialization, and motor. In the current study, the VABS Communication Domain was used to assess language ability, including expressive, receptive, and literacy skills. The standardized score for each domain was used.

#### Symptom Severity of ASD

The Autism Diagnostic Observation Schedule (ADOS-G) is a semi-structured play- and conversation-based assessment. Module 1, 2, or 3 was administered depending on the child's level of expressive language. The revised algorithm and calibrated severity scores described by Gotham and colleagues (Gotham et al. 2006) were used because they are better suited than the original algorithm scores for comparing ADOS scores across modules. The revised algorithm calculates ADOS summary scores for social and repetitive behavior domains. The calibrated severity scores are less influenced by age and language level than are raw scores (Gotham et al. 2008).

### Statistical Analyses

Pearson's chi-squared test (when appropriate) were used to ascertaining between-group differences concerning parent characteristics and age of children. To compare children with ASD with and without core ASD symptoms at 18 months to non-ASD children with and without core ASD symptoms at 18 months, on cognition, functional language and ASD symptom severity, a dummy variable (4 groups) was created combining ASD diagnosis (yes/no) and risk status at 18 months (yes/no). Children in the four groups were compared on (a) cognition (IQ), (b) functional language (VABS), and (c) ASD symptom severity (ADOS-CSS) by conducting a one-way ANOVA with Bonferroni correction for post-hoc analyses (see Table 1). All statistical analyses were performed using SPSS 26.

**Table 1:** Child test characteristic across child diagnosis and screening status on DFA6 from M-CHAT

	GROUP	Children without ASD		Children with ASD		F	P
	DFA6	N = 671		N = 163			
		A N = 612	B N = 59	C N = 116	D N = 47		
		Below cut off	Cut off or higher	Below cut off	Cut off or higher		
Mean (SD)							
IQ^a^		100.2 (14.1)	73.2 (20.7)	87.9 (20.4)	60.2 (15.3)	133.75*	< .001
VABS^b^ functional language	95.9 (12.5)	73.2 (15.4)	78.4 (17.2)	61.0 (13.2)	165.54*	< .001	
ADOS^c^ CSS	1.4 (1.1)	2.7 (2.2)	5.2 (2.5)	6.1 (2.7)	284.10*	< .001	

All differences significant except group B vs. C on VABS functional language

^a^WASI, SB-5 or Mullen scales of early development

^b^Vineland Adaptive Behaviour Scale—communication domain

^c^Autism diagnostic observation schedule 4 N in each group varies due to missing data on outcome measures (from 1 to 8 children missing)

## Results

Of the 163 children diagnosed with ASD in the ABC study/NPR linkage, 47 (28.8%) met cut-off or above on DFA6 at 18 months according to early ASD symptoms (at-risk, group D) compared to 116 children with ASD scoring below cut-off according to early ASD symptoms (low-risk, group C). Comparable numbers in the non-ASD group were 59 (8.8%) who met cut-off on DFA6 at 18 months (at-risk, group B) compared to 612 children in the non-ASD group scoring below the cut-off (low-risk, group A). There were significant between-group differences in the proportion of children reaching cut-off on DFA6 across age and sex (see Table 2). In all four groups (A to D), the majority reaching cut-off were boys. The ratio of boys to girls in the ASD groups (C, D) was approximately 7: 1 in the low-risk group and approximately 1.5: 1 in the at-risk group. The comparable ratio in the non-ASD groups (A, B) was 1.5: 1 in the low-risk group and approximately 5.0: 1 in the at-risk group.

**Table 2:** Child age and sex across child diagnosis and screening status on DFA6 from M-CHAT

	Group	Children without ASD		Children with ASD		F	P
	DFA6	N = 671		N = 163			
		A N = 612	B N = 59	C N = 116	D N = 47		
		Below cut off	Cut off or higher	Below cut off	Cut off or higher		
Age at assessment	3 and 4 years	587 (95.9%)	57 (96.6%)	60 (51.7%)	40 (85.1%)	211.488^a^	< .001
	5 and 6 years	13 (2.1%)	2 (3.4%)	17 (14.7%)	3 ( 6.4%)		
	7 to 10 years	12 (2.0%)	0 (0.0%)	39 (33.6%)	4 (8.5%)		
Sex	Male	367 (60.0 &)	49 (83.1%)	101 (87.1%)	30 (63.8%)	40.318 ^4^	< .001
	Female	245 (40.0%)	10 (16.9%)	15 (12.9%)	17 (36.2%)		

^a^Fisher’s exact test (expected number in cells < 5)

There were no significant between-group differences related to maternal age, maternal education, or parity (see Table 3). Subsequent analyses were conducted on all children (with/without ASD) regardless of risk status at 18 months. This was done to identify child characteristics, possibly contributing to the determination of risk status.

**Table 3:** Parental characteristics across child diagnosis and symptom status on DFA6 from M-CHAT

Total N = 834	Group	Children without ASD		Children with ASD		Test statistics	P value
	DFA6	N = 671		N = 163			
		A N = 612	B N = 59	C N = 116	D N = 47		
		Below cut off^a^	Cut off or higher	Below cut off ^0^	Cut off or higher		
Maternal age	< 25 years	57 (9.3%)	6 (10.2%)	15 (12.9%)	2 (4.3%)	χ^2^ = 13.916	= .125
	25–29 years	185 (30.2%)	12 (20.3%)	39 (33.6%)	22 (46.8%)		
	30–34 years	240 (39.2%)	28 (47.5%)	46 (39.7%)	16 (34.0%)		
	> 34 years	130 (21.2%)	13 (22.0%)	16 (13.8%)	7 (14.9%)		
Maternal education	< 12 years^b^	42 (6.9%)	8 (13.6%)	7 (6.0%)	6 (12.8%)	χ^2^ = 11.607	= .478
	12 years^c^	153 (25.0%)	14 (23.7%)	38 (32.8%)	13 (27.7%)		
	13–16 years^d^	241 (39.4%)	24 (40.7%)	44 (37.9%)	16 (34.0%)		
	> 17 years^e^	160 (26.1%)	11 (18.6%)	25 (21.6%)	12 (25.5%)		
	Missing	16 (2.6%)	2 (3.4%)	2 (1.7%)	0 (0.0%)		
Parity	0	281 (45.9%)	23 (39.0%)	67 (57.8%)	25 (53.2%)	χ^2^ = 7.839	= .049
	1+	331 (54.1%)	36 (61.0%)	49 (42.2%)	22 (46.8%)		

Fisher’s exact test or Pearson’s Chi-squared test

^a^Rated with none or only one ASD symptom

^b^Not completed high school

^c^High school level

^d^Bachelor level

^e^Master/Ph.D. level

### Cognition

Analysis of IQ score at the time of clinical assessment indicated a significant association between 18 months risk status (at-risk/low-risk according to whether early ASD symptoms were present or absent) and later cognitive skills (F (3, 816) = 133,749, p < 0.001). Post-hoc analysis revealed that children with at-risk status at 18 months had lower IQ scores at the time of assessment compared to low-risk children, both in the ASD group and non-ASD group. The mean difference in IQ between children below DFA6 cut-off compared to those scoring at cut-off or above were M_diff_ = 27.7, CI95% (20.2, 35.2) IQ points in the ASD group, and M_diff_ = 26.9, CI95% (21.0, 32.7) in the non-ASD group. Children diagnosed with ASD and scoring below cut-off on DFA6 at 18 months (low-risk) had a mean IQ score at the assessment of M = 87.9, CI95% (84.0, 91.7) compared to children with ASD who scored at or above cut-off with M = 60.2, CI 95% (55.7, 64.7). This indicates an association between showing core ASD symptoms (at-risk) at 18 months and a lower cognitive level (IQ measure) at the time of assessment.

### Functional Language

Analyses using standardized scores of functional language (VABS) at the time of assessment indicated a significant association of risk status (at-risk/low-risk on presence/absence of early ASD symptoms) and later functional language skills (F (3, 795) = 165,537, p < 0.001). Post-hoc analysis revealed that children with at-risk status at 18 months, in general, had lower standardized VABS functional language scores at the time of assessment compared to low-risk children, both in the ASD group and non-ASD group. The mean difference in functional language score between children scoring below DFA6 cut-off compared to those scoring at cut-off or above was M_diff_ = 17.4, CI95% (11.2, 23.7) in the ASD group, and M_diff_ = 22.7, CI95% (17.7, 27.7) in the non-ASD group. Children diagnosed with ASD and scoring below cut-off on DFA6 at 18 months (low-risk) had a mean functional language score at the assessment of M = 78.4, CI95% (75.1, 81.6) compared to children with ASD who scored at or above cut-off with M = 61.0, CI95% (55.7, 64.7). This indicates an association between showing core ASD symptoms at 18 months (at-risk) and lower functional language skills (VABS) at the time of assessment.

### ADOS Calibrated Severity Score (ADOS CSS)

Analyses of ADOS symptom severity score at the time of assessment indicated a significant association between risk status (at-risk/low-risk on presence/absence of early ASD symptoms) and later symptom severity (F (3, 825) = 284,101, p < 0.001). Post-hoc analysis revealed that children with at-risk status at 18 months, had higher symptom severity scores at the time of assessment compared to low-risk children, both in the ASD group and non-ASD group. The mean difference in symptom severity score between children scoring below DFA6 cut-off compared to those scoring at cut-off or above were M_diff_ = − 1.0, CI95% (− 1.7, − 0.2) in the ASD group and M_diff_ = − 1.3, CI95% (− 1.8, − 0.7) in the non-ASD group. Children diagnosed with ASD and scoring below cut-off on DFA6 at 18 months (low-risk) had a mean symptom severity score at the assessment of M = 5.2, CI95% (4.7, 5.6) compared to children with ASD who scored at or above cut-off with M = 6.1, CI 95% (5.3, 6.9). This indicates an association between showing core ASD symptoms at 18 months (at-risk) and higher symptom severity (ADOS-CSS) at the time of assessment.

### Other Diagnoses

The DFA6 did not capture the majority of children at 18 months who were diagnosed with ASD at the clinical assessment in the ABC study or from the NPR registry. Thirty percent of the children diagnosed with Autistic Disorder (non-syndromic) did show ASD symptoms at 18 months (at-risk), compared to 22% of those later diagnosed with Asperger syndrome or PDD-NOS at the ABC clinic or in NPR. Seven children identified with ASD in the clinic had a concurrent known syndrome combined with severe intellectual disability (N = 5) or had a history of loss of skills (disintegrative disorder) (N = 2). The two children with loss of skills scored, as expected, below cut-off on DFA6 at 18 months, and the other five ("syndromic ASD") scored at or above cut-off on the DFA6 criterion. To further explore the characteristics of children identified by the DFA6, we also looked at the distribution (below cut-off/above cut-off on DFA6) across other relevant diagnoses in the sample, e.g., specifically children diagnosed with language impairment or with intellectual disability (Table 4).

**Table 4:** Diagnostic result across screening status on DFA6 from M-CHAT

N (%)	Below cut-off	Cut-off or higher	Total
No Dx or ClinProbl	288	3 (1.0%)	291
Autistic disorder	65	28 (30.1%)	93
PDD-Nos	49	14 (22.2%)	63
Syndrome ASD or loss of skills	2	5 (71.4%)	7
Intellectual disability (ID) no ASD	6	26 (81.3%)	32
Language disorder (LD) noASD noID	165	23 (4.4%)	188
Other Dx or ClinProbl	153	7 (4.4%)	160
Total	728	106	834

*Dx* diagnosis, *ClinProbl* clinical problems, *ID* intellectual disability, *ASD* autism spectrum disorder, *LD* language disorder

The clinical sample comprised of 32 children with a primary diagnosis of Intellectual Disability. Most of these children (N = 26; 81.3%) scored above the six critical item criteria (DFA6). However, in the group of children with a primary diagnosis of Language Impairment at assessment, only approximately one in twenty children (N = 23; 4.4%) scored at or above the DFA6 cut-off at 18 months. In children with no diagnoses or clinical problems, only three scored at or above the six critical item criteria (DFA6) of two or more core ASD symptoms at 18 months (N = 3, 1.0%).

## Discussion

The present study compared intellectual level, functional language skills and ASD symptom severity at the age of diagnostic assessment (age range 3–10 years) with ASD at-risk status at 18 months of age based on the symptom score on the six critical items of the M-CHAT (DFA6) and diagnostic outcomes in the ABC study/NPR (ASD, non-ASD). The results indicate an association between parents' recognition of clinical signs of ASD at 18 months (at or above cut-off on DFA6) and the severity of cognitive impairments in children with ASD at diagnostic assessment. Furthermore, children with ASD who showed core ASD symptoms at 18 months (at-risk) scored lower on functional language skills at the clinical assessment and with increased symptom severity (ADOS CSS) score compared to children with ASD who scored below cut-off on DFA6 (low-risk) at 18-months, as is also true for the non-ASD groups as well.

The finding that children with ASD who scored below the cut-off for ASD symptoms (DFA6) at 18 months scored within the average IQ range at assessment, and children with ASD who scored at or above the cut-off for core ASD symptoms at 18 months scored within the intellectual disability range, is consistent with previous reports. Early identification of ASD is associated with general developmental delay and more pronounced cognitive impairment than later identified children, suggesting that a more significant impairment is necessary to raise parental concerns as early as 18 months, or that early symptoms of ASD might be more heterogeneous than those often considered prototypical for ASD (Baghdadli et al. 2003; Chawarska et al. 2006, 2014; Giacomo & Fombonne 1998; Guthrie et al. 2019; Iverson et al. 2019; Øien et al. 2018a; Shattuck et al. 2009). Several population-based studies show that a large proportion of children who later were diagnosed with ASD were not identified by screening instruments at 18 months (Baird et al. 2000; Guthrie et al. 2019; Øien et al. 2018a; Stenberg et al. 2014). It is likely that population-based samples, in contrast to clinical samples (or stage 2 screening), include more children who are within the average range of IQ and functional language skills. Children referred for clinical assessment are likely to be more severely affected and have more prototypical symptoms than children in a general population-based sample. Additionally, in clinical samples, parents are already likely to express concern about their child's development, which might influence how they answer questionnaires and rate symptom patterns. In line with this, commonly used screening instruments seem to perform more ‘accurately' in high-risk samples (i.e., children referred for diagnostic assessment) than in low-risk samples (children attending routine visits to well-baby clinics) (Kleinman et al. 2008; Pandey et al. 2008). This suggests that the instruments are picking up children with ASD who have lower intellectual and adaptive functioning while missing children with more robust general intellectual development and better functional language (Eaves et al. 2016; Kamio et al. 2013; Øien et al. 2018b; Salomone et al. 2015; Snow and Lecavalier 2008). In other words, the performance of screening instruments at an early age, as reported in many high-risk clinical samples, seems biased towards more severe behavioral characteristics of ASD and other atypical developmental phenomena.

Difficulties in identifying children with ASD early in the developmental period are related to the substantial heterogeneity that characterizes ASD (Ozonoff et al. 2010; Zwaigenbaum et al. 2015), affecting both the screening and the diagnostic process. Still, research suggests that ASD can be reliably diagnosed very early (Lord et al. 2006) and that early diagnoses of ASD are stable (Chawarska et al. 2007; Ozonoff et al. 2015). Another contributing factor is the possibility that a large proportion of children developing ASD do not show the prototypical signs of ASD at 18 months. ASD is a developmental disorder, and symptoms often emerge gradually. It is not known whether a different set of descriptors better captures key characteristics in children missed by current instruments or if the behavioral characteristics of these children are more subtle and thus cannot meet actionable concern. Such challenges make it difficult to construct an appropriate checklist with the salient symptom description relevant at an early age for the whole spectrum of children with ASD (Øien, Schjølberg, et al. 2018a, b). There are indications that children need to show more significant impairment to be identified by screening instruments (Baghdadli et al. 2003; Giacomo and Fombonne 1998; Shattuck et al. 2009). One possibility is that subgroups of children with ASD might show differences in developmental trajectories throughout the first years of life, both in symptom pattern and in heterogeneity in age when features of autism begin to manifest and in a variation of when social demands exceed the child's capabilities (Chawarska et al. 2006). The findings of the present study suggest that future research should focus on mapping the developmental trajectories of children with the aim of understanding factors that contribute to a child’s at-risk or low-risk status at various times in early development. Further, future research should aim to relate these findings to long-term outcome developmentally, behaviorally and diagnostically. Such knowledge might lead to the development of new methods and insights in early identification of children with lower symptom severity and cognitive impairment and to a better understanding of developmental processes underlying the emergence of autism features in higher-functioning children with ASD at an early age. It is possible that broader developmental measures (e.g. motor development and temperament) might prove useful and eventually contribute to a better understanding of the variability in early trajectories and hence contribute to a decrease in the age of diagnosis across the whole spectrum.

Findings from this study and other studies (Guthrie et al. 2019; Øien et al. 2018a; Stenberg et al. 2014; Yuen et al. 2018) warrant a meaningful discussion on the feasibility of universal screening at 18 months and give directions for moving to capture different phenotypical variants of autism spectrum disorder at an early age. In this study, the early at-risk score was defined at 18 months based on the six critical items from M-CHAT and was related to diagnostic conclusions several years later. It might be necessary to revisit our determination of prototypical early developmental features of ASD. One strategy for follow-up would be to utilize a broad set of assessment tools to capture characteristics and changes in behavior patterns across age and to look specifically at the developmental trajectories of children with ASD who have preserved language and intellectual abilities. The fact that some of these children are identified early, both by parents and professionals, should bring forward opportunities to study their development in greater detail. At present, studies indicate that finding one single measure to identify all ASD-relevant developmental profiles of delayed and deviant behavior in early childhood might be utopian at this point, and that such measures might be close to or impossible to construct due to the heterogeneous nature of ASD. However, it is essential to follow the same goal-directed course as Robins and Colleagues on the M-CHAT and the later M-CHAT-R (Robins et al. 2001, 2014). Over almost two decades, they have provided the field with the measures that currently –in spite of their shortcomings– are regarded as the gold standard of screening instruments. In the end, the best approach to early identification may be to combine multiple measures and to consider parental concern as “red-flags,” even when concern for prototypical signs of ASD are not present. As Mick Jagger and Keith Richards sing: “You can’t always get what you want” might also apply to the possibility of developing one measure to identify all children with ASD. It may be necessary to envision a gold standard, in terms of psychometric properties, for screening instruments for early detection as relative rather than absolute (Øien et al. 2019).

### Strengths and Limitations

Previous studies in the field of early identification of children with ASD have mostly used clinical samples and retrospective data collection. The principal strength of this study is prospective data collection and information about signs of ASD before the diagnosis has been established. The MoBa cohort is population-based; thus, both children with mild and severe ASD symptoms are included in the study.

Only six items on ASD characteristics at 18 months were used for the analyses in the present study. In a previous study, we found that the three M-CHAT items that contributed to the highest likelihood ratios for ASD (the proportion of screen-positives among ASD cases relative to the proportion of screen-positives among non-cases) were among these six items that had been found in other studies to be most critical for distinguishing ASD (Stenberg et al. 2014).

Most population-based studies are affected by selection bias. Compared with the Norwegian birth cohort, the MoBa cohort and the ABC Study cohort have an under-representation of young mothers (< 25 years), mothers who have single status, mothers who smoked during pregnancy, and non-users of prenatal folic acid supplements (Nilsen et al. 2009, 2013).

Different methods were used to assess intellectual level due to the age span and different levels of intellectual ability in our sample. This is not optimal because different IQ measures are normed on different samples. However, the IQ difference between at-risk and low-risk 18-month-old children in the current study was so substantial that we would not expect this to be an effect of the different IQ measures in of itself. All IQs were calculated based on US norms because Norwegian norms were not yet available for WASI, SB-5, or Mullen.
